# Age-Related Differences in Functional and Structural Connectivity in the Spatial Navigation Brain Network

**DOI:** 10.3389/fncir.2019.00069

**Published:** 2019-10-29

**Authors:** Stephen Ramanoël, Elizabeth York, Marine Le Petit, Karine Lagrené, Christophe Habas, Angelo Arleo

**Affiliations:** ^1^Sorbonne Universités, INSERM, CNRS, Institut de la Vision, Paris, France; ^2^Centre for Clinical Brain Sciences, University of Edinburgh, Edinburgh, United Kingdom; ^3^Normandie Université, UNICAEN, PSL Université Paris, EPHE, INSERM, U1077, CHU de Caen, Neuropsychologie et Imagerie de la Mémoire Humaine, Caen, France; ^4^CHNO des Quinze-Vingts, INSERM-DHOS CIC 1423, Paris, France

**Keywords:** healthy aging, MRI, resting-state, diffusion, connectivity, spatial navigation, vision

## Abstract

Spatial navigation involves multiple cognitive processes including multisensory integration, visuospatial coding, memory, and decision-making. These functions are mediated by the interplay of cerebral structures that can be broadly separated into a posterior network (subserving visual and spatial processing) and an anterior network (dedicated to memory and navigation planning). Within these networks, areas such as the hippocampus (HC) are known to be affected by aging and to be associated with cognitive decline and navigation impairments. However, age-related changes in brain connectivity within the spatial navigation network remain to be investigated. For this purpose, we performed a neuroimaging study combining functional and structural connectivity analyses between cerebral regions involved in spatial navigation. Nineteen young (μ = 27 years, σ = 4.3; 10 F) and 22 older (μ = 73 years, σ = 4.1; 10 F) participants were examined in this study. Our analyses focused on the parahippocampal place area (PPA), the retrosplenial cortex (RSC), the occipital place area (OPA), and the projections into the visual cortex of central and peripheral visual fields, delineated from independent functional localizers. In addition, we segmented the HC and the medial prefrontal cortex (mPFC) from anatomical images. Our results show an age-related decrease in functional connectivity between low-visual areas and the HC, associated with an increase in functional connectivity between OPA and PPA in older participants compared to young subjects. Concerning the structural connectivity, we found age-related differences in white matter integrity within the navigation brain network, with the exception of the OPA. The OPA is known to be involved in egocentric navigation, as opposed to allocentric strategies which are more related to the hippocampal region. The increase in functional connectivity between the OPA and PPA may thus reflect a compensatory mechanism for the age-related alterations around the HC, favoring the use of the preserved structural network mediating egocentric navigation. Overall, these findings on age-related differences of functional and structural connectivity may help to elucidate the cerebral bases of spatial navigation deficits in healthy and pathological aging.

## Introduction

Spatial navigation represents one of the most fundamental activities of daily life and it requires the integration of multiple processes. These processes include the perception of spatial information from a variety of sensory cues, the creation and maintenance of spatial representations in memory, and the manipulation of these representations to guide navigational behavior (Wolbers and Hegarty, 2010; Julian et al., 2018). These spatial navigation abilities are mediated by numerous cerebral regions across the brain (Burgess, 2008; Spiers and Barry, 2015). These cerebral regions encompass interconnected structures of the medial temporal lobe such as the hippocampus (HC) and parahippocampal cortices, along with distal areas including the retrosplenial, posterior-parietal and prefrontal cortices. These brain structures relevant to spatial navigation are involved in diverse ways depending on the spatial representation strategy used. The medial temporal lobes, including the HC and entorhinal cortex, are predominantly activated for allocentric (world-centered) representations, whereas the posterior parietal regions tend to support egocentric (self-centered) strategies (Burgess et al., 2002; Herweg and Kahana, 2018). The medial prefrontal cortex (mPFC) plays a key role in maintaining useful information in working memory and in selecting the most appropriate navigational strategy for the complexity of the task at hand (Wolbers et al., 2007; Spiers and Gilbert, 2015; Chrastil et al., 2017; Ito, 2018).

Given the advanced nature of the human visual system, successful human spatial navigation requires the careful integration of complex high-resolution visual information (Ekstrom, 2015). A growing body of work has shed light on the importance of three high-level visual regions for the integration of relevant visual information for navigation—namely, the parahippocampal place area (PPA), the retrosplenial cortex (RSC), and the occipital place area (OPA; Vann et al., 2009; Julian et al., 2018). The PPA, located close to the parahippocampal and the lingual gyri, plays a role in landmark processing (Janzen and van Turennout, 2004; Epstein, 2008), such as 3D geometric structures (Henderson et al., 2008) and spatial boundaries (Park et al., 2011). The PPA appears to be involved in contextual association (Marchette et al., 2015) and place recognition (Epstein and Vass, 2014) during navigation tasks. Lesions to the RSC, located posteriorly to the corpus callosum, produce topographical disorientation but they may also transiently impair visual memory (Maguire, 2001). The RSC is thought to play a role in general scene processing, stability of landmarks (Auger et al., 2012; Auger and Maguire, 2018) and viewpoint integration—particularly with regards to translation of information between egocentric and allocentric representations (Vann et al., 2009; Mitchell et al., 2018). Finally, the function of the OPA, located near the intraparietal sulcus, remains unclear but its hypothesized roles include the representation of environmental boundaries (Julian et al., 2016) and the integration of discrete views in a 360° environment (Robertson et al., 2016). Furthermore, two recent studies have demonstrated the capacity of the OPA to encode potential future pathways during spatial navigation (Bonner and Epstein, 2017; Patai and Spiers, 2017). Although the PPA, RSC and OPA appear to be crucial for high-level visual processing related to spatial navigation, these scene-selective regions are also sensitive to low-visual features in scenes including dominant cardinal orientations (Nasr and Tootell, 2012), spatial frequencies, and contrast (Kauffmann et al., 2015).

All these cerebral areas, which process visual information and encode spatial representations, form the spatial navigation network. Healthy aging has a deleterious impact on this neural network, which manifests itself as navigational impairments, reducing the autonomy of older adults (Moffat, 2009; Lithfous et al., 2013; Lester et al., 2017). Several studies have reported a specific decline in the use of allocentric strategies in navigation tasks (for a review, see Klencklen et al., 2012). This is linked to the age-related atrophy and dysfunction of hippocampal regions, as well as to age-dependent changes occurring in the parahippocampal gyrus, the RSC, and the frontal regions (Moffat et al., 2006; Zhong and Moffat, 2018). On the other hand, egocentric strategies, which are supported by parietal and striatal regions, appear to be relatively well preserved in older adults (Harris and Wolbers, 2012; Harris et al., 2012). In addition, recent neuroimaging studies have highlighted age-related changes within low-visual regions dedicated to central visual field processing (Brewer and Barton, 2012; Ramanoël et al., 2015). A decline in low-level visual processing is expected to negatively influence spatial navigation.

To date, the majority of work investigating the age-related neurocognitive decline in navigation abilities has focused on brain regions engaged during spatial navigation separately. Less consideration has been given to the navigation network as a whole. Magnetic resonance imaging (MRI) is a useful tool for non-invasively studying the age-related differences in functional and structural connectivity between regions within the navigation network. Functional connectivity can be probed using resting-state functional MRI, which measures the correlation between low-frequency spontaneous fluctuations in the blood oxygen level dependent (BOLD) signal across brain regions in the absence of an explicit stimulus (Damoiseaux, 2017). The absence of a specific task in resting-state paradigms is advantageous in the study of healthy aging as it reduces confounding variables related to skill level, experience and fatigue. Structural connectivity can be evaluated using diffusion-weighted MRI. This technique is sensitive to the Brownian motion of water molecules within a given voxel of the brain, and, in particular, within the constraints of white matter tracts, hence allowing tract alterations to be studied. More precisely, this technique measures the general diffusion of water molecules related to barriers and obstacles imposed by the arrangement of fibers and cell membranes in brain tissue (for a detailed description of diffusion-weighted imaging methods, see Jones et al., 2013; Wandell, 2016; Jeurissen et al., 2017). Different parameters of interest can be extracted from which the white matter fiber organization can be inferred (Soares et al., 2013). The fractional anisotropy quantifies the diffusion direction preference, and it is particularly sensitive to microstructure changes like axonal density (Mori and Zhang, 2006; Lerner et al., 2014). The axial and radial diffusivity enable the characterization of diffusivity in the principal diffusion axis and in both secondary axes, respectively. Both axial and radial diffusivities are sensitive to changes in myelin and axonal integrity in healthy subjects (Kumar et al., 2013; Winklewski et al., 2018).

Aging induces a reorganization of the functional and structural connectivity of the brain. A decrease in connectivity within several cerebral networks, including the default-mode network and the executive network, is a common finding of whole brain functional studies and it is associated with age-related cognitive decline (for a review, see Ferreira and Busatto, 2013). With respect to structural connectivity, recent studies using diffusion imaging have reported a decrease in fractional anisotropy associated with an increase in mean and radial diffusivities in normal aging (Bennett and Madden, 2014). While there is evidence for functional and structural connectivity changes in normal aging, such effects in the spatial navigation brain network remain poorly studied. To our knowledge, only Korthauer et al. (2016) have investigated age-related structural connectivity changes within the spatial navigation network. In this study, participants performed a computerized version of the hidden-platform Morris water-maze task. Results from this study showed a fractional anisotropy decrease in the uncinate fasciculus (a white matter tract connecting the HC with the mPFC) amongst older adults, which was associated with an increased time delay when solving the task. However, only whole brain analysis was performed as opposed to network analysis of structural connectivity between those brain structures that are specifically dedicated to spatial navigation. Furthermore, with the exception of the RSC, brain structures involved in high-level visual information processing during human spatial navigation were not considered.

The present study evaluated the effect of normal aging on the spatial navigation brain network. To accomplish this aim, we focused on how functional and structural connectivity differ between young and older groups within key structures involved in navigation: the mPFC, the HC, and the scene-selective regions (PPA, RSC, and OPA). In addition, considering the crucial role of vision for human spatial navigation, we additionally included the projection into the visual cortex of central and peripheral visual fields in our navigation network analysis. We anticipated age-related differences in the spatial navigation network in functional and structural connectivity. We hypothesized that there would be heterogeneous connectivity changes between key regions of interest within our spatial navigation network, and that such changes would be associated with spatial cognition, in particular regarding navigational strategies.

## Materials and Methods

### Participants

Among the 41 healthy participants initially enrolled in the study, two older participants were excluded due to anatomical abnormalities, which resulted in failure of the normalization procedure. The participants were part of the French cohort population SilverSight (~350 subjects) established and followed-up ever since 2015 at the Vision Institute—Quinze-Vingts National Ophthalmology Hospital, Paris. All participants were native French speakers, and they gave their written informed consent to participate in the study. All screening and experimental procedures were in accordance with the tenets of the Declaration of Helsinki and they were approved by the Ethical Committee “CPP Ile de France V” (ID_RCB 2015-A01094-45, CPP N°: 16122). All participants had normal or corrected-to-normal vision, and they had no history of neurological or psychiatric disorders, or sensorimotor dysfunctions. Older participants had a score of 26 or higher on the Mini Mental State Examination (MMSE; Folstein et al., 1975). The ensemble of clinical and functional examinations used to enroll the participants included: an ophthalmological and functional visual screening, a neuropsychological evaluation, an oculomotor screening, an audio-vestibular assessment as well as a static/dynamic balance examination. The neuropsychological assessment included a computerized version of the 3D mental rotation test (3D-Rotation, Vandenberg and Kuse, 1978), the perspective-taking test (PTT, Kozhevnikov and Hegarty, 2001), the Corsi block-tapping task (Corsi, 1972), and the figural memory test (FGT, short and long) implemented in the Vienna test system software (Schuhfried, 2012).

### MRI Acquisition

Images were acquired using a 3T Siemens Magnetom Skyra whole-body MRI system (Siemens Medical Solutions, Erlangen, Germany) with a 64-channel head coil at the Quinze-Vingts National Ophthalmology Hospital in Paris, France. Resting-state functional magnetic resonance imaging (fMRI), diffusion-weighted imaging and an anatomical image were acquired for all participants, followed by two additional functional localizer runs. The anatomical volume consisted of a T1-weighted, high-resolution, three-dimensional MPRAGE sequence (TR/TE/IT/flip angle = 2,300 ms/2.9 ms/900 ms/9°; matrix size = 256 × 240 × 176; voxel size = 1 × 1 × 1.2 mm). For the resting-state functional scan, 304 volumes of 60 slices were acquired using a T2*-weighted simultaneous multi-slice echo planar sequence (SMS-EPI; TR/TE/flip angle = 1,000 ms/30 ms/90°; matrix size = 96 × 96; SMS = 2; GRAPPA = 2; voxel size = 2.5 mm isotropic). Diffusion-weighted images were acquired along 128 gradient directions (TR/TE/flip angle = 1,600 ms/84 ms/90°; 14 volumes *b* = 0 and 128 volumes *b* = 1,500 s/mm^2^, SMS = 2; GRAPPA = 4; voxel size = 2.5 mm isotropic). For the functional localizer scans of scene-selective regions and low-visual areas, 364 and 304 volumes respectively of 64 slices were acquired using a T2*-weighted SMS-EPI sequence (TR/TE/flip angle = 1,000 ms/30 ms/90°; matrix size = 100 × 100; SMS = 2; GRAPPA = 2; voxel size = 2.5 × 2.5 × 2.4 mm).

### Stimuli and Procedure

Two independent functional localizers were used to map individually scene-selective regions (PPA, RSC, OPA) and the projection into visual cortex of central and peripheral visual fields (CVF, PVF). A block fMRI paradigm, adapted from Ramanoël et al. (2015) was then employed to locate scene-selective areas. Participants were presented with blocks of gray scale photographs (256 gray scales), all sized 900 × 900 pixels (or 18 × 18 degrees of visual angle) for scenes, faces, everyday objects, scrambled scenes and scrambled objects. The functional run lasted 6 min and it was composed of 14, 20 s task blocks (four blocks of scenes, four blocks of scrambled scenes, two blocks of faces, two blocks of objects and two blocks of scrambled objects), including 20 different images of the same category, and interspersed with four block of 20 s each, with a fixation dot in the center of the screen displayed against a gray background. Each stimulus was presented for 400 ms followed by a 600 ms inter-stimulus interval with a fixation dot in the center of the screen. Participants performed a “one-back” repetition detection task.

To define ROIs for CVF and PVF regions, we used a blocked eccentricity mapping experiment adapted from Chang et al. (2015). Two rings composed of a black and white checkboard flickering at 4 Hz were sequentially presented at the center (2° eccentricity) for 15 s and at the periphery (8° eccentricity) for 15 s of the visual field. All participants completed one 5 min functional run. To ensure that participants remained focused on the central point, they were asked to respond with a button press when they saw a red or a green cross on the fixation dot. During the resting-state fMRI acquisition, participants were asked to close their eyes, to not think about anything in particular and to remain awake.

### MRI Data Pre-processing and Statistical Analyses

#### Definition of the Navigation Network (ROIs)

Processing of localizer data was performed using SPM12 release 7,487 (Wellcome Department of Imaging Neuroscience, London, UK^1^) implemented in MATLAB 2018a (Mathworks Inc., Natick, MA, USA). For each participant, the first four functional localizer volumes were discarded to allow for equilibration effects and the remaining images were realigned to correct for head movements and co-registered to the T1-weighted anatomical image. The images were analyzed using a single participant general linear model. Slice-timing correction was not applied in line with the recommendations of the Human Connectome Project functional pre-processing pipeline for multi-slice sequences (Glasser et al., 2013). Similarly to Ramanoël et al. (2018), a study-specific template in MNI space was created using Diffeomorphic Anatomical Registration Exponentiated Lie algebra (DARTEL) to improve inter-subject alignment during normalization (Ashburner and Friston, 2005; Ashburner, 2007). Finally, functional scans were smoothed with a 6 mm full-width half maximum (FWHM) Gaussian kernel.

Statistical analysis was performed using general linear model (Friston et al., 1995) at single participant level. For each participant, eight conditions of interest (scenes, faces, objects, scrambled scenes, scrambled objects, fixation, center ring and peripheral ring) were modeled as eight regressors, constructed as box-car functions and convolved with a canonical hemodynamic response function. Movement parameters obtained from realignment corrections were also considered in the model as an additional factor of no interest. Time-series for each voxel were high-pass-filtered (1/128 Hz cut-off) to remove low-frequency noise and signal drift.

PPA, RSC and OPA regions were located independently for each participant as ROIs using the fMRI contrast [Scenes > (Faces + Objects)]. CVF and PVF areas were mapped using the contrasts (Center > Periphery) and (Periphery > Center), respectively. Significant voxel clusters on individual t-maps were identified using false discovery rate correction (FDR) for multiple comparisons (alpha = 0.05) to control for the overall false-positive rate. Sphere ROIs (5 mm radius) were created at individual peaks of activation in each scene-selective and low-visual region and in each cerebral hemisphere (individual peak coordinates for PPA, RSC, OPA, CVF and PVF are available in the Supplementary Table S1).

For the hippocampal region, participant-specific ROI masks for the left and right HC were created using the VolBrain online brain volumetry software (Manjón and Coupé, 2015,^2^). Furthermore, ROI masks for the mPFC in the right and left hemispheres were obtained from the automated anatomic labeling atlas (AAL; Tzourio-Mazoyer et al., 2002).

#### Pre-processing and Statistical Analysis of Functional Connectivity Data

Resting-state pre-processing steps were similar to those for localizer paradigms described above. In addition, the Artifact Detection Toolbox, included within the CONN functional connectivity toolbox^3^ (Whitfield-Gabrieli and Nieto-Castanon, 2012), was used to detect scans with excessive movement. The resulting scrubbing parameters were added as a first-level covariate, along with realignment parameters (six parameters obtained by rigid body correction of head motion). Physiological and other spurious sources of noise were estimated using CompCor (Behzadi et al., 2007), and they were regressed out together with white matter, cerebrospinal fluid, motion parameters, age and gender covariates. Linear detrending and band-pass filtering (0.008–0.09 Hz) were carried out during regression. Correlation analyses were performed by extracting the mean BOLD signal time series from ROIs and computing Pearson’s correlation coefficients between subject-specific ROIs including the PPA, RSC, OPA, CVF, PVF, HC, and the mPFC for each hemisphere and each group. We applied Fisher’s r-to-z transformation to improve the normality of the bivariate correlation matrix for each participant (14 × 14). To investigate the age-related difference in functional connectivity, two-sided two-sample *t*-tests were then performed to compare ROI-to-ROI connectivity between the young and the older group. The results were considered significant at *p* < 0.05 FDR corrected for multiple comparisons and effect sizes were evaluated with Hedges’ g score.

#### Pre-processing and Statistical Analysis of Diffusion Data

All diffusion processing steps were performed using functions implemented in FSL (FMRIB Software Library^4^). Diffusion MRI data were first pre-processed to remove eddy-current-induced distortions and motion artifacts. Brain tissues, were extracted using the Brain Extraction Tool. Diffusion images were visually inspected after each processing step to control for obvious artifacts. Maps of diffusion parameters (including fractional anisotropy, FA, mean diffusivity, MD, axial diffusivity, AD, and radial diffusivity, RD) were calculated using DTIfit. A tract-based ROI-to-ROI approach was taken for diffusion analyses. The diffusion tensors were fitted using the Bayesian Estimation of Diffusion Parameters Obtained using Sampling Techniques (BEDPOSTX), which models fibers crossing in each voxel.

The resulting parameters were used to reconstruct the distribution probability of tract locations using probabilistic tractography (PROBTRACKX). This approach considers crossing-fiber pathways and it provides maps of the probability of the presence of a fiber in a voxel (Jbabdi and Johansen-Berg, 2011; Soares et al., 2013). This probabilistic technique includes a parameter which accounts for the number of tracts between the seed ROI (i.e., the starting point) and the target ROI (i.e., the destination) divided by the total number of tracts from the seed ROI. The connection between the seed and the target depends on other tracts than those included in the seed-target pathway. The probability of connection from the seed to the target can differ from the probability of connection from the target to the seed (Wandell, 2016). Therefore, each navigational ROI, after being registered to the diffusion space, was used consecutively as a seed and as a target. This enabled the creation of a map for each combination of anatomical connectivity between two ROIs. Five thousand samples per voxel were generated, a curvative threshold of 0.2 was used and a correction for the distance across seed and target regions was applied. Each tracking image was normalized to MNI space using linear and nonlinear registration tools (FLIRT and FNIRT) and divided by the total number of samples generated creating a proportion image. Those maps were also divided by the total number of tracts in order to account for cross-subject “tractability” differences. Participants’ proportion maps were merged to create a mean group image with a threshold fixed at *p* < 5.10E-6 to generate a mean mask of tracts that were not rejected by the selection criteria. Group masks were then restricted to voxels for which FA value was superior to 0.2. Mean masks of each tract were used as restriction masks on individual proportion maps to extract mean diffusion parameters (FA, MD, AD, RD) for each pair of ROIs and each hemisphere (14 × 14).

Despite the correction, the probability of connectivity between two ROIs decreases as a function of distance (Morris et al., 2008). In cases where the mPFC was used as a seed, the signal was lost after thresholds were applied. Hence, the anatomical connectivity of this region was not considered here.

Differences in mean diffusion parameters between young and older groups in the new matrix (12 × 12) were assessed using a two-sided two-sample *t*-test and they were considered significant for *p* < 0.05 corrected for multiple comparisons [*p* = 0.05/(12 × 12 × 4 × 2)]. Similarly to the functional connectivity analysis, gender was included as a covariate and effect sizes were evaluated with Hedges’ g score. Mean diffusion parameters were not averaged across tracts however using two regions as seed and target (Wandell, 2016). This allowed more accurate results to be obtained and it provided confirmation that tracts in these two sides showed the same diffusion parameter direction differences between groups.

## Results

MRI data were analyzed from 39 participants divided into two age groups: a young group (*N* = 19; 10 females; mean age ± SD: 27 ± 4.3 years; age range: 21–37 years) and an older group (*N* = 20; 10 females; mean age ± SD: 73 ± 4.1 years; age range: 66–80 years). All subjects had visual, oculomotor, and audio-vestibular faculties in between or above normative age-dependent limits. In Table 1, we present the behavioral performances obtained in the cognitive tests for the older group only. Cognitive data for the young participants were not included here for the partial dataset of neuropsychological assessment for young participants (12 of 19 young participants completed the entire neuropsychological evaluation).

**Table 1:** Mean and standard deviation (SD) of scores obtained on cognitive tests in the old group; 3D mental rotation test (3D-Rotation), perspective-taking test (PPT), short and long-term figural memory test (FGT-short and FGT-long), short and long Corsi block-tapping task (Corsi-short and Corsi-long).

	Old group	
Gender (M/F)	10/10	
	Mean	SD
3D-Rotation	11.2	2.9
PTT	45.5	26.4
FGT-short	5.8	1.8
FGT-long	6.2	1.9
Corsi-short	4.4	0.8
Corsi-long	4.4	0.8

### Age-Related Differences in Functional Connectivity in the Spatial Navigation Network

**Figure 1:**
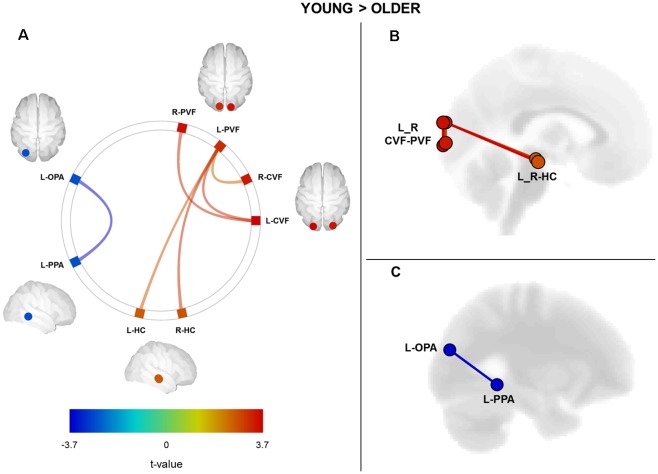
Cerebral regions (ROI-to-ROI analysis) showing a significant difference between groups (Young Group vs. Older Group) in functional connectivity. **(A)** Connectome ring with navigational brain regions showing a decrease (in red) and an increase of functional connectivity for older adults compared to young ones. **(B)** Sagittal view of the brain with regions showing a significant decrease of functional connectivity in older adults. **(C)** Sagittal view of the brain with regions showing a significant increase in functional connectivity in older subjects. The statistical significance threshold was set at *p* < 0.05 FDR-corrected for two-sided analysis. L, left; R, right; CVF, central visual field; PVF, peripheral visual field; OPA, occipital place area; PPA, parahippocampal place area; HC, hippocampus.

We performed a ROI-to-ROI functional connectivity analysis on the resting-state fMRI data by computing the strength of correlation between all possible pairs of ROIs within the navigation network. We sought age-related differences by directly comparing ROI-to-ROI correlation values between young and older groups (Figure 1). We found a significant age-related decrease in the functional connectivity between visual cortical fields and the HC (*p* < 0.05, FDR correction for multiple comparisons; see Supplementary Table S2 for full details on statistical correlations). More precisely, older adults exhibited a decrease in the functional connectivity between the left peripheral visual field region and the right and left HC as compared to the young group (Figures 1A,B). Associated with these changes between visual and hippocampal regions, we found a significant increase in the functional connectivity between OPA and PPA regions in the left hemisphere of older adults (Figures 1A,C; see Supplementary Table S2 for detailed statistics). Finally, we did not find any significant age-related differences in functional connectivity for the RSC and the mPFC regions.

Hedges’g scores for the effect size of significant differences between young and older participants ranged from 0.8 to 1.1. As proposed by Cohen (1988), the magnitude of the effects reported here was considered large (effect size > 0.8).

### Age-Related Differences in Structural Connectivity in the Spatial Navigation Network

**Figure 2:**
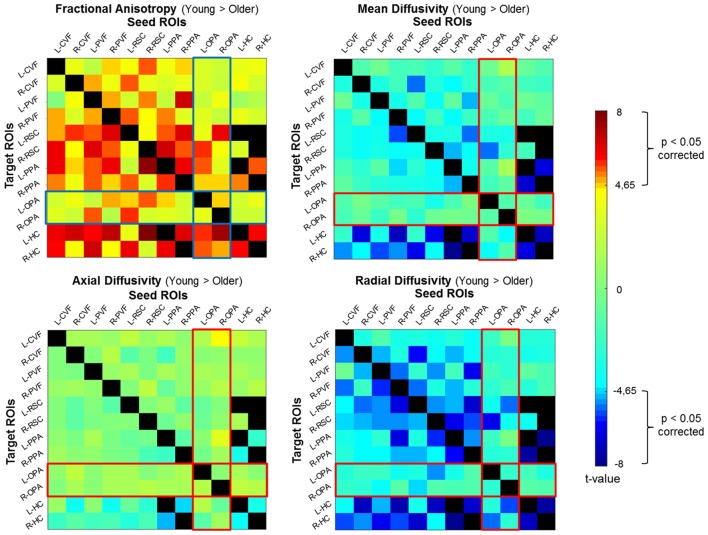
Correlation matrices representing differences between groups (Young Group vs. Old Group) for diffusion parameters. The statistical significance threshold was set at *p* < 0.05 corrected for multiple comparisons for two-sided analysis. FA, fractional anisotropy; MD, mean diffusivity; AD, axial diffusivity; RD, radial diffusivity.

We conducted a probabilistic tract-based ROI-to-ROI analysis on diffusion data to assess the structural connectivity within the navigation network. We evaluated age-related differences by comparing ROI-to-ROI diffusion values for each diffusion parameter (fractional anisotropy, mean diffusivity, axial diffusivity, and radial diffusivity) between groups (Figure 2). We found age group differences with a significant decrease in fractional anisotropy and an increase in mean and radial diffusivity for older adults as compared to young participants across all ROIs (see Supplementary Table S3 for full statistical details and Supplementary Figure S1). More precisely, we reported significant age-related structural connectivity differences between all ROIs for at least one diffusion parameter. Interestingly, we also found that the anatomical connectivity around the OPA region was more preserved in older adults with respect to other navigational ROIs (with the exception of the connectivity between the OPA and RSC). The ROI-to-ROI tracts showing age-related differences in our spatial navigation network matched with three main tracts from the JHU-white matter tractography atlas: the posterior part of the inferior longitudinal fasciculus (ILF), the posterior part of the inferior fronto-occipital fasciculus (IFOF) and the forceps major.

**Figure 3:**
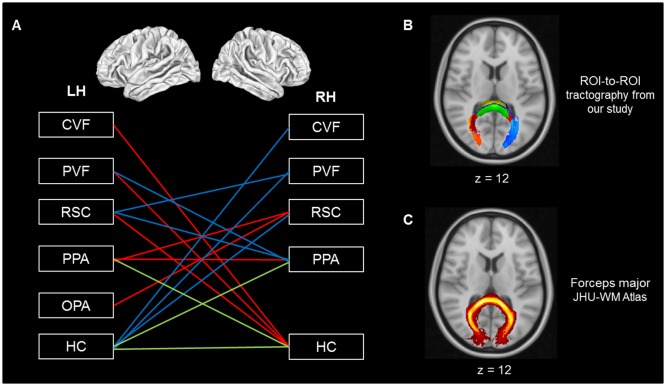
**(A)** Cerebral regions showing covariation of diffusion parameters between hemispheres. **(B)** Tractography from our analysis showing a covariation of FA, MD and RD parameters for young compared to older adults. **(C)** Forceps major tract from the JHU white-matter tractography atlas (Hua et al., 2008). Red line: connection from LH to RH, blue line: connection from RH to LH, green line: both hemispheres, LH, left hemisphere; RH, right hemisphere; CVF, central visual field; PVF, peripheral visual field; OPA, occipital place area; PPA, parahippocampal place area; RSC, retrosplenial cortex; HC, hippocampus. FA, fractional anisotropy; MD, mean diffusivity; RD, radial diffusivity; YG, young group; OG, old group.

We then examined the co-variation of fractional anisotropy, the mean and radial diffusivities between age groups. Results revealed differences in structural connectivity only between both hemispheres in the navigation network (Figure 3A). We found age-related differences between the left low-visual areas and the right HC (red lines), as well as between the right low-visual areas and the left HC (blue lines). We also found structural age-related connectivity differences between the right and the left HC and between HC and PPA for both hemispheres (green line). Interestingly, the ROI-to-ROI tracts from our analysis showing a covariation of fractional anisotropy, mean and radial diffusivities for young adults compared to older subjects (Figure 3B), which appeared to be very similar to the forceps major tract from the JHU white-matter tractography atlas (Figure 3C; Hua et al., 2008). As expected, the connectivity matrices were asymmetric (see Supplementary Figure S1). We found similar age-related diffusion parameter differences when ROIs were considered as seed and target, with the exception of the HC. Indeed, differences were only seen for the HC as a target ROI, but not as a seed. This result could be explained by the correction applied during the normalization of maps related to the size of the seed ROI.

The magnitude of the effects for structural results reported here was between 1.1 and 2.2 (> 0.8 considered large).

### Association Between Neuropsychological Assessment and Connectivity Measures

We conducted correlational analyses between cognitive scores obtained from the neuropsychological evaluation (3D-Rotation, PPT, FGT short and long, Corsi short and long) and connectivity measures (functional and structural) for the older group only. We found no significant association between functional connectivity and cognitive scores. By contrast, for structural connectivity, we showed a significant correlation between fractional anisotropy values and two pairs of ROIs within our navigational network. More precisely, we found a negative correlation (*r* = −0.87) for the 3D-Rotation test between the R-CVF and the R-OPA; and a positive correlation (*r* = 0.80) for the FGT (long) between the R-OPA and the R-RSC [*p*-values were Bonferroni-corrected for multiple comparisons: *p* = 0.05/(12 × 12 × 6)].

Finally, we conducted a correlation analysis between functional and structural brain connectivity measures for all participants. Results showed no significant correlations.

## Discussion

The present neuroimaging study assessed age-related brain connectivity differences within the spatial navigation network. Our results indicate that both functional and structural connectivity of brain areas involved in spatial coding and navigation are altered in healthy aging. Our functional results show decreased connectivity between low-visual areas and the HC, associated with an increase in connectivity between OPA and PPA in older participants compared to young subjects. Concerning anatomical connectivity, we observed a general decline in white matter integrity of the spatial navigation network in older adults. We found altered anatomical connectivity in several ROIs including low-visual areas, scene-selective regions and the HC, characterized by a modification of fractional anisotropy, as well as mean and radial diffusivities. In addition, the relative lack of differences in OPA connectivity suggests that the structural connectivity of this region is less disrupted than that of other regions within the spatial navigation network.

### Functional Connectivity

From a functional perspective, we found age-related differences in the connectivity between low-level visual areas and high-level spatial areas. First, we showed a decrease in functional connectivity within the posterior part of our network, encompassing CVF and PVF between both hemispheres and dedicated to processing low-visual information in older adults. Age-related functional connectivity changes between low-visual regions could be interpreted in light of subtle visual deficits (e.g., decreases in visual acuity and contrast sensitivity) typically seen in normal aging (Owsley, 2011, 2016) and related brain changes. Healthy older adults, for example, show a reduced surface area and increased population receptive field sizes in the foveal representations of V1, V2 and hV4 compared to young adults (Brewer and Barton, 2012, 2014). In addition, previous studies highlighted specific visual deficits in normal aging related to low-visual activation within early visual areas and scene-selective regions (Ramanoël et al., 2015). Second, our results showed an age-related decrease in functional connectivity between low-visual areas and the HC, a key structure within the navigation network. Several studies on healthy subjects (Chadwick et al., 2013; Zeidman et al., 2015) and on patients with hippocampal lesions (Lee et al., 2005) have demonstrated the role of the HC in visual perception. For example, Chadwick et al. (2013) reported a top-down influence of the HC on early visual areas. Our results, showing a decreased functional connectivity associated with aging, do not allow us to determine the direction of effect between top-down modulation (i.e., reactivation of sensory representations) and bottom-up modulation (i.e., visuospatial input leading to representations of visual environment).

Conversely, the functional connectivity was higher for older compared to young participants in the anterior part of the spatial navigation network, between the left OPA and left PPA. The role of PPA in spatial navigation has previously been described in relation to the representation of landmark-based navigation (Epstein and Vass, 2014). The HC, on the other hand, is considered to provide the neural basis for the encoding of spatial information and long-term memory (see Maguire and Mullally, 2013 for a review). The PPA is able to sufficiently support the recognition of scenes and landmarks, however, without the need for engagement of the HC (Köhler et al., 2002; Epstein, 2008). Furthermore, the parahippocampal regions are less affected by normal aging than the HC itself (Zhong and Moffat, 2018). The increased functional connectivity between the OPA and the PPA in normal aging may, therefore, represent a putative compensatory mechanism in spatial navigation as these regions assume greater navigational roles in order to counter the reduced connectivity of the HC. Previous work has suggested that the PPA may be functionally divided into two distinct scene-processing networks (Baldassano et al., 2016). Of note is the posterior PPA association with the OPA as a visual network. The increased connectivity between the OPA and PPA in the present study may hence represent visual components in aging. Alternatively, the anterior-posterior distinction of the PPA may disappear with age, as the anterior PPA assumes a role similar to the HC.

Contrary to previous research, we did not observe an age-related difference in functional connectivity of the RSC. Moffat et al. (2006), for example, demonstrated that older participants exhibited reduced fMRI activation in the posterior HC, posterior parahippocampal gyrus and RSC compared to younger participants when encoding a virtual environment. Again, our focus on functional connectivity at rest rather than task-related activity may be the source of such discrepancies.

### Structural Connectivity

Concerning anatomical connectivity, our results showed a general decline in white matter integrity of the spatial navigation network in older adults, in line with previous studies using a whole-brain MRI diffusion approach (Fjell et al., 2009; Bennett and Madden, 2014; Coupé et al., 2017). These results, in conjunction with the absence of change for the axial diffusivity parameter, suggest an age-related loss of white matter integrity within the spatial navigation network, which is likely to reflect demyelination rather than axonal loss (Madden et al., 2009; Bennett et al., 2010). Interestingly, we found a relative lack of differences in OPA connectivity suggesting that the structural connectivity of this region is less disrupted than that of other regions within the spatial navigation network.

The consideration of the covariation of diffusion parameters in our structural connectivity analyses permits further interpretation of our results. The covariation of multiple diffusion metrics within a given tract, compared to single-factor variation, aids the biological interpretation of white matter structural changes (Soares et al., 2013). As such, when solely ROIs presenting fractional anisotropy, mean and radial diffusivities co-varying connectivity differences between older and young groups are considered, the apparent inter-hemispheric nature of these differences is noteworthy. Moreover, these connectivity differences between hemispheres involve a unique white matter tract, namely the forceps major located on the splenium of the corpus callosum. The forceps major connects the occipital lobes and it plays an important role in processing visual cues (Goldstein and Mesfin, 2017). Since axial diffusivity remains unmodified, white matter modifications of this tract during aging appear to be related to demyelination. This result is consistent with previous studies showing a decrease in the structural integrity of the forceps major during aging (Bennett and Madden, 2014). Furthermore, this tract seems to be largely involved in spatial navigation. For example, Tamura et al. (2007) reported the case of a patient who developed similar symptoms to pure topographical disorientation after experiencing a lesion within the right forceps major. More specifically, this patient presented with visuospatial deficits and difficulty using information derived from landmarks. Diffusion modifications observed in the forceps major during aging in our study are consistent with the interhemispheric disconnection model proposed by O’Sullivan et al. (2001), thus suggesting that disconnection plays a role in cognitive deficits with aging.

### Behavioral Relevance of Age-Related Connectivity Differences

Altogether, our structural and functional findings suggest an age-related decline in the connectivity of the pathway from low-visual areas to the HC, combined with a maintained connectivity around the OPA. These results may be interpreted as a compensatory mechanism for age-related alterations around the HC, favoring the use of the preserved structural network in the region of the OPA mediating egocentric navigation. This pattern agrees with the current literature regarding spatial navigational strategies in aging. The HC, and in particular the hippocampal body, is known to be vulnerable to normal aging (Malykhin et al., 2017). Volume reductions of the HC have been associated with allocentric spatial navigation deficits (Guderian et al., 2015; Colombo et al., 2017), and there is a tendency for greater reliance on egocentric as opposed to allocentric spatial navigation strategies with increasing age (Harris et al., 2012; Rodgers et al., 2012; Gazova et al., 2013). The OPA, on the other hand, plays a key role in egocentric spatial navigation strategy, related to visual information (Bonner and Epstein, 2017). For example, this high-level visual region appears to be sensitive to egocentric distance and to first-person perspective motion (Kamps et al., 2016; Persichetti and Dilks, 2016).

Moreover, the age-related differences reported here are also involved in the visual cortex. Visual information is crucial for successful navigation (Ekstrom, 2015). Several studies have emphasized a key interaction between the HC and areas involved in visual perception during the internal representation of spatially coherent scenes (Zeidman et al., 2015; Zeidman and Maguire, 2016). In the past, age-related changes in spatial cognition have generally been attributed to poor strategic choice and memory loss due to frontal and hippocampal atrophy. The impact of a decline in visuo-perceptual ability with age has tended to be neglected and future studies should consider the impact of such a decline. This is particularly relevant in light of the distinct visual scene-processing network, composed of the OPA and PPA, described by Baldassano et al. (2016). The age-related changes in the posterior regions of the spatial navigation network in the present study adhere to the classic posterior-to-anterior shift theory of healthy aging (Davis et al., 2008). However, Cabeza et al. (2018) recently proposed greater clarification of the terminology surrounding compensation mechanisms in healthy aging. In particular, Cabeza et al. (2018) differentiated between compensation by selection, by upregulation and by reorganization. As the current study focused on functional connectivity at rest and structural connectivity only, it is not possible to know precisely which compensation mechanism is engaged. Contrary to our expectations, one of our findings showed a negative correlation between fractional anisotropy values between the CVF and OPA and 3D-Mental Rotation scores for older participants. In other words, greater fractional anisotropy in normal aging was associated with a decrease in mental visual rotation abilities. Future work could clarify this last result and the nature of the compensation mechanism, perhaps by using task-based functional MRI to measure BOLD response within the spatial navigation network during a task aimed at testing allocentric and egocentric navigation strategies.

Concerning the involvement of the RSC in the spatial navigation network, our results show an age-related loss of structural white matter integrity around the RSC, but no changes in functional connectivity of this high-level visual brain region. Several studies have highlighted the key function of the RSC in spatial navigation (for a review, see Mitchell et al., 2018) such as its role in translating from egocentric into allocentric reference frames. Neuroimaging studies have reported that the RSC activity was related to recollection processing of permanent visual landmark during a navigation task using a first-person perspective (Auger et al., 2012; Auger and Maguire, 2018). This point was partially assessed by our results showing that older participants with fractional anisotropy values close to young participants between the OPA and RSC exhibited better performance on the figural memory test. Concerning RSC-PPA connectivity, a recent study showed that functional and anatomical changes in a patient suffering from developmental topographic disorientation were associated with spatial navigation impairments (Kim et al., 2015). However, in the present study of healthy aging, we did not observe functional connectivity differences within the RSC-PPA pathway. The preservation of functional connectivity here in healthy older adults may account for their relatively spared navigational abilities compared to patients with topographic disorientation. Indeed, several studies have suggested that a loss of white matter integrity leads to functional connectivity changes (Ferreira and Busatto, 2013).

Surprisingly, regarding the anterior component of the spatial navigation network, there were no specific age-related changes in functional and structural connectivity between the mPFC and other ROIs in the spatial navigation network. Previous work has nonetheless shown white matter damage to be more pronounced in anterior compared to posterior regions (Gunning-Dixon et al., 2009; Madden et al., 2009), as well as between the HC and the mPFC (Korthauer et al., 2016). The divergence of our results from current literature could be partially explained by methodological differences. In the majority of MRI studies, diffusion data is acquired in each phase-encoding direction in order to correct image distortions, which mainly appear in frontal regions. In our work, with the aim of minimizing the MRI examination duration for older participants, diffusion data were acquired in solely one direction. Moreover, in our probabilistic tractography analysis, a conservative threshold was applied. These two factors are associated with a bias related to the longer distance between mPFC and all other ROIs, which could explain the absence of results from the mPFC in the spatial navigation brain network. As such, caution is advised regarding the results from the frontal regions of the spatial navigation brain network.

### Limitations

The findings of the present study should be considered in light of several limitations. Here, we focused solely on certain key visual structures within the spatial navigation network. Previous studies have, however, considered additional cerebral areas such as prefrontal, motor and cerebellar regions or the entorhinal cortex (Doeller et al., 2008; Rodriguez, 2010; Iglói et al., 2014; Hao et al., 2016; Stangl et al., 2018). Further research using task-based fMRI involving different navigation strategies, combined with a complete neuropsychological assessment of navigation skills, would permit the validation of the inclusion and/or exclusion of key structures within the spatial navigation network as well as the investigation of brain connectivity related to visual information processing for navigation. Notably, a recent computational modeling study (Bicanski and Burgess, 2019) highlighted the importance of considering entorhinal cortex connectivity with frontal and parietal regions to investigate age-related changes in visuospatial abilities. Furthermore, this would address difficulties regarding the interpretation of functional connectivity changes. For example, increases in functional connectivity could represent a disruption rather than an improvement related to behavior. An additional limitation of the present study is the lack of information regarding the dynamic nature of connectivity changes in normal aging. Longitudinal studies of normal aging would provide a means to evaluate this issue. Finally, future studies should consider investigating the hippocampal subfields as proposed by Hrybouski et al. (2019, see also Dalton et al., 2019) to further elucidate age-related changes in the spatial navigation network.

## Conclusion

To conclude, the present study sheds light on specific age-related differences in connectivity between key cerebral structures involved in spatial navigation. The older adults enrolled in this study exhibited a decrease in functional connectivity between low-visual areas and the HC, associated with greater functional connectivity between OPA and PPA regions. Interestingly, structural connectivity results showed differences in white matter integrity within the navigation brain network, although less pronounced around the OPA. This pattern of brain connectivity differences can be interpreted as a compensatory mechanism for the age-related alterations concerning the HC, thus favoring the use of the preserved structural network mediating egocentric navigation. These findings emphasize the importance of considering the link between the decline of visual and navigation abilities in future studies on the effects of normal or pathological aging in spatial cognition.

## Data Availability Statement

The datasets generated for this study are available on reasonable request to the corresponding author.

## Ethics Statement

The studies involving human participants were reviewed and approved by Ethical Committee “CPP Ile de France V” (ID_RCB 2015-A01094-45, CPP N°: 16122). The patients/participants provided their written informed consent to participate in this study.

## Author Contributions

SR, CH and AA: conceived the study. SR, EY, MP and KL: data acquisition. SR, EY and MP: data processing. SR, EY, MP and AA: manuscript writing.

## Conflict of Interest

The authors declare that the research was conducted in the absence of any commercial or financial relationships that could be construed as a potential conflict of interest.

## Footnotes

^1^www.fil.ion.ucl.ac.uk/spm/

^2^http://volbrain.upv.es/

^3^https://web.conn-toolbox.org/

^4^http://www.fmrib.ox.ac.uk
