# Supplementary figures and images for: Age-Related Differences in Functional and Structural Connectivity in the Spatial Navigation Brain Network
Source: Front Neural Circuits. 2019 Oct 29;13:69. doi: 10.3389/fncir.2019.00069 (PMC6828843; doi:10.3389/fncir.2019.00069)

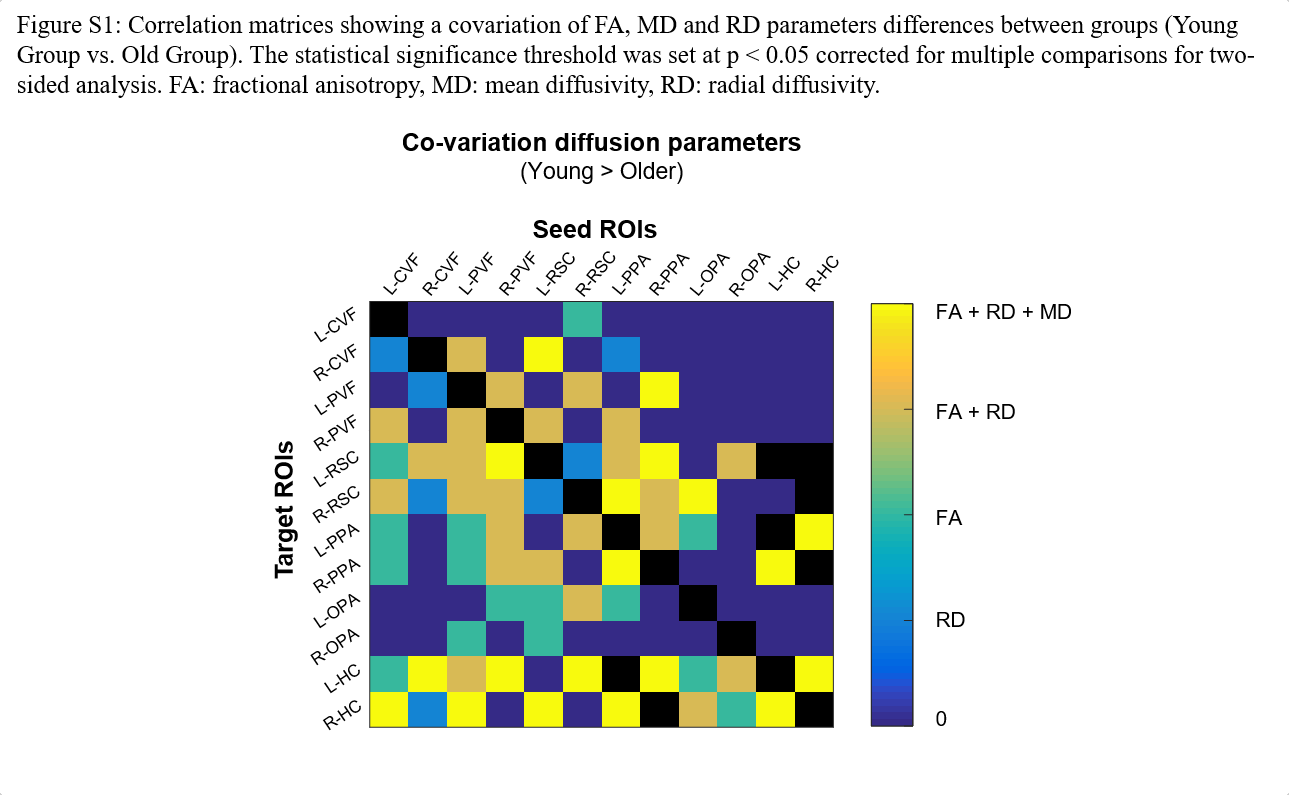

Supplement: Supplementary file 4 [file Image_1.tif]
